# Onset matters in dementia with Lewy bodies

**DOI:** 10.18632/aging.204730

**Published:** 2023-05-11

**Authors:** Sebastian Johannes Müller, Jens Wiltfang, Niels Hansen

**Affiliations:** 1 Department of Neuroradiology, University Medical Center Goettingen, Germany; 2 Department of Psychiatry and Psychotherapy, University Medical Center Goettingen, Germany; 3 German Center for Neurodegenerative Diseases (DZNE), Germany; 4 Neurosciences and Signaling Group, Institute of Biomedicine (iBiMED), Department of Medical Sciences, University of Aveiro, Portugal; 5 Translational Psychoneuroscience, University Medical Center Goettingen, Germany

**Keywords:** dementia with Lewy bodies, psychiatric-onset of prodromal dementia with Lewy bodies, magnetic resonance imaging

Although the prevalence of dementia involving Lewy bodies constitutes 4% of all dementia cases in the population and 7.5% in secondary care [1], the disease remains underdiagnosed. The main diagnostics criteria are clinical or based on additional biomarkers [2]. Early diagnosis is important to optimize health management. Three different forms of prodromal dementia with Lewy bodies have recently been proposed, namely (1) onset with psychiatric symptoms, (2) onset with mild cognitive impairment (MCI), and onset with delirium not attributable to other causes of delirium [3]. MRI currently plays a minor role as a supportive biomarker. The typical pattern is mild global brain atrophy accompanied by marked moderate gray matter loss in the dorsal midbrain, substantia innominata, and hypothalamus [4]. In Alzheimer's disease, the atrophy of these regions is even more pronounced.

In our study, we first manually and automatically (using FastSurfer) analyzed specific atrophy patterns in a neuropsychiatric cohort of patients with DLB and a control cohort using 1.5 and 3 Tesla MRI [5]. We observed mild to moderate atrophy of the whole brain involving accentuation of the amygdala, hippocampus, and right pars orbital cortex. A moderately atrophic substantia innominata was also apparent. Inspired by finding that local atrophy in the right transverse superior temporal lobe was detectable in a subset of DLB patients revealing poor color vision [6], we diversified our patients according to their clinical data. In the second step [7], we divided these patients into two subgroups according to their disease’s onset: mild cognitive impairment (*n* = 30) and psychiatric episodes (*n* = 30). Patients with delirium (*n* = 3) were excluded. Automated volumetry via a standard atlas (Desikan-Kiliany-Tourville DKTatlas) revealed no significant differences between the two groups. Manual measurements (Figure 1) revealed significantly more extensive atrophy of the left substantia innominata (*p* = .018) in patients with a psychiatric onset than in those with an onset of mild cognitive impairment. Additional subgroup analysis revealed even more pronounced substantia-innominata atrophy in patients whose onset of dementia with Lewy bodies was psychiatric in nature.

**Figure 1:**
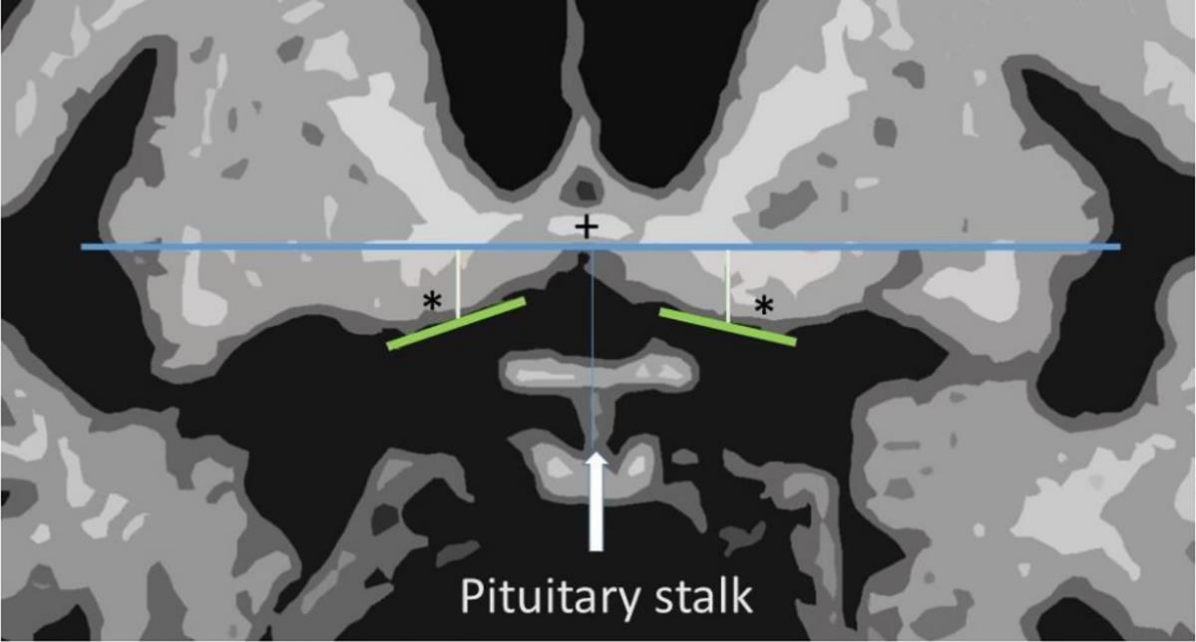
**Schematic drawing of the measurement method.** + - anterior commissure; * - nucleus basalis of Meynert; blue line - horizontal line under the anterior commissure; green line - line under nucleus basalis of Meynert; white orthogonal line- measured distances.

This finding is inconsistent with published study data and may indicate progressive atrophy in this brain region during the disease course, eventually leading to psychiatric symptoms. We may have made this observation because of the fact that this was a neuropsychiatric rather than neurological cohort. On the other hand, it could also be a random finding due to methodological-measurement shortcomings or retrospective data. Volumetric evaluations of frontobasal regions remain difficult, especially in retrospective studies involving heterogeneous MR image data. Special issues such as innominata atrophy require optimized segmentation algorithms and atlases.

Over the last few years, improvements have been made in automated MRI analysis involving segmentation methods and volumetric analysis of brain areas. Of particular note are the open-source FreeSurfer and FastSurfer projects [8] employing high-resolution, T1-weighted sequences. These methods are being constantly improved, and there are dozens of packages offering specialized segmentation and labeling algorithms now being developed.

At the same time, MR technology is also getting better, and existing methods continue to improve. Images produced by the new 7 Tesla MRI scanners offer higher resolution. Methods to quantify imaging data (e.g., for T1 relaxation time values) developed theoretically as early as the 1990s, are now used in everyday clinical practice thanks to modern computing power. Here, new methods are available to us in the short and long term to more precisely study the exact anatomy and pathophysiology of dementia and brain atrophy *in vivo*, especially prodromal dementia with Lewy bodies. However, it will also enable us to better and more accurately quantify natural aging and brain regression in conjunction with the clinical phenotype of patients to determine future biomarkers to enable the early diagnosis of dementia with Lewy bodies as a prominent form of alpha-synucleinopathy.

## References

[1] Vann Jones SA, et al. Psychol Med. 2014; 44:673–83. 10.1017/S0033291713000494 23521899

[2] McKeith IG, et al. Neurology. 2017; 89:88–100. 10.1212/WNL.0000000000004058 28592453 PMC5496518

[3] McKeith IG, et al. Neurology. 2020; 94:743–55. 10.1212/WNL.0000000000009323 32241955 PMC7274845

[4] Whitwell JL, et al. Brain. 2007; 130:708–19. 10.1093/brain/awl388 17267521 PMC2730778

[5] Khadhraoui E, et al. BMC Neurol. 2022; 22:114. 10.1186/s12883-022-02642-0 35331168 PMC8943955

[6] Unger RH, et al. J Alzheimers Dis. 2019; 72:1233–40. 10.3233/JAD-190727 31683482

[7] Hansen N, et al. Front Aging Neurosci. 2022; 14:815813. 10.3389/fnagi.2022.815813 36274999 PMC9580213

[8] Henschel L, et al. Neuroimage. 2020; 219:117012. 10.1016/j.neuroimage.2020.117012 32526386 PMC7898243

